# Family planning use and its associated factors among women in the extended postpartum period in Addis Ababa, Ethiopia

**DOI:** 10.1186/s40834-017-0054-5

**Published:** 2018-01-05

**Authors:** Almaz Yirga Gebremedhin, Yigzaw Kebede, Abebaw Addis Gelagay, Yohannes Ayanaw Habitu

**Affiliations:** 1 Pathfinder International, Addis Ababa, Ethiopia; 2 Department of Epidemiology and Biostatistics, Institute of Public Health, College of Medicine and Health Sciences, University of Gondar, Gondar, Ethiopia; 3 Department of Reproductive Health, Institute of Public Health, College of Medicine and Health Sciences, University of Gondar, Gondar, Ethiopia

**Keywords:** Postpartum period, Family planning, Kolfe Keranyo, Addis Ababa, Ethiopia

## Abstract

**Background:**

Postpartum period is an important entry point for family planning service provision; however, women in Ethiopia are usually uncertain about the use of family planning methods during this period. Limited studies have been conducted to assess postpartum family planning use in Addis Ababa, in particular and in the country in general. So, this study was conducted to assess postpartum family planning use and its associated factors among women in extended postpartum period in Kolfe Keranyo sub city of Addis Ababa.

**Materials and methods:**

A community-based cross sectional study was conducted from May to June 2015 on 803 women who have had live births during the year (2014) preceding the data collection in the sub city. The multi-stage cluster sampling technique was used to select study participants. Data were collected by interviewer administered structured questionnaire, entered into EPI INFO version 7 and analyzed by SPSS Version 20. Bivariable and Multivariable logistic regression models were employed to see the presence and strength of the association between the dependent and independent variables by computing the odds ratios with a 95% confidence intervals and *p*-values.

**Results:**

The prevalence of postpartum family planning use was 80.3% (95% CI: 74.5, 83.1). Marriage, (AOR 0.09, 95% CI: 0.03, 0.22), menses resumption after birth, (AOR 2.12, 95% CI: 1.37, 3.41), length of time after delivery, (AOR 2.37, 95% CI: 1.18, 4.75), and history of contraceptive use before last pregnancy, (AOR 0.12, 95% CI: 0.07, 0.18) were the factors associated with postpartum family planning use.

**Conclusion:**

The prevalence of postpartum family planning use was high and the main factors associated with it were marriage, menses resumption, length of time after delivery, and history of previous contraceptive use. Therefore women should get appropriate information about the possibility of exposure to pregnancy prior to menses resumption by giving special emphasis to those who had no previous history of contraceptive use and exposure to the other identified factors.

## Background

Family planning (FP) is an essential component of health care provided during the antenatal period, immediately after delivery, and during the first postpartum year [1]. Postpartum family planning (PPFP) is defined as the prevention of unintended pregnancy and closely spaced pregnancies during the first 12 months following childbirth [1]. The promotion of family planning in countries with high birth rates can avert 32% of all maternal deaths and nearly 10% of childhood deaths [2]. Though family planning can avert that much maternal and childhood deaths, postpartum fertility and contraception are generally not well understood by policymakers, health service providers, or the women themselves [3]. Hence, promoting and providing PPFP is a vital issue as it saves the lives of mothers and children [1, 3, 4].

Evidences showed that the use of family planning was low among postpartum women in spite of their unmet need for family planning [3]. There are two groups of PPFP methods, namely traditional and modern [5–7]. The traditional methods of PPFP include breastfeeding, abstinence, the calendar, and lactational amenorrhea [7]’ While, modern methods involve intrauterine contraceptive devices (IUCD), implants (Implanon, Jadelle, sinoplant), injectables, progesterone-only oral contraceptives, coils, and condoms [7]. The effectiveness of the two groups of family planning methods (Modern versus Traditional) is not equal in that the failure rate of traditional methods is high [7]. The recommended time for the initiation of contraceptives in the postpartum period is 6 weeks after delivery [8]. Short and long pregnancy intervals have risks on perinatal outcomes, like increased risks of preterm birth, low birth weight, and small-for-gestational age [9, 10]. All these evidences suggest that spacing pregnancies appropriately could help prevent such adverse perinatal outcomes and that PPFP use is of paramount importance.

The prevalence of contraceptive use among postpartum women varies from region to region in Ethiopia, as most women do not start taking contraceptives at the recommended time [11]. Even those who use PPFP rely on traditional, mainly lactational amenorrhea (LAM) that might pose the risk of unintended pregnancy. Therefore, initiating appropriate contraception in the postpartum period is important to avoid negative health outcomes.

Research conducted in Istanbul showed that only 34.0% of mothers began contraceptive methods 5 months after childbirth [12]. Generally, women and family members did not perceive birth spacing as a priority, as women who deliver most recently were not using contraception [12, 13]. Another study conducted in Gondar showed that 48.8% of mothers used PPFP [14].

A variety of literature showed that factors like maternal age [15, 16], employment status [17], religion or culture [4, 18–21], lack of awareness of family planning methods [14, 20, 22], male involvement [4, 18, 21], extended family [18, 23], death of child [17], antenatal care follow up [24], inaccessibility of family planning methods [18, 20], and fear of side effects [18] were some of the factors affecting PPFP use among women in the postpartum period [4].

Therefore, by considering the above situation, this study set out to assess postpartum family planning use and its associated factors among women in extended postpartum period in Kolfe Keranyo sub-city, Addis Ababa.

## Methods

### Study design

A community-based cross-sectional study design was employed to obtain data from women who had live births 12 months prior to the survey.

### Study period and study area

This study was conducted from May to June 2015 in Kolfe Keranyo sub-city which is located south-west of Addis Ababa. The sub-city is divided into 15 administrative areas (districts). According to the 2014 population projection estimates, there were 500,163 residents in the sub-city, with half of them being women [13]. In addition, there were twelve health centres, 2 health posts and no hospital at Kolfe Keranyo sub-city.

### Source and study population

The source population of this study was women who had live births 12 months prior to the survey with the exception of those who were unable to respond during the survey in Kolfe Keranyo sub- city.

### Sample size determination and sampling procedure

The sample size was determined using the single population proportion formula, considering the following assumptions: Prevalence (P) of family planning use during postpartum period = 52.5% [13], margin of error (w) =5%, design effect of 2, 10% non response rate, Z_α/2_ = 1.96 at 95% confidence interval. The total sample calculated was 849.

A multistage cluster sampling technique was used to select the participants. First, out of the fifteen districts of Kolfe Keranyo sub-city, four were chosen by the simple random sampling technique (lottery method). Considering proportion, sixteen ketanas (smallest administration units of sub-cities) were selected using the lottery method. Then, the total sample size was distributed proportionally to each cluster (ketana). Postpartum women in the selected ketenas were interviewed through house to house visits until the predetermined sample size allocated to each cluster was completed. The data were collected using an interviewer administered questionnaire by ten BSc Graduate nurses who had previous experience in data collection, and the process was supervised by two experts who had Master’s degree in Public Health and previous experience in research supervision. The collected data were checked for completeness daily.

### Data quality assurance

To assure the reliability and validity of the questionnaire, a pre-test was conducted on 44 individuals living outside the study area. Training was given to data collectors and supervisors for 1 day before data collection.

### Operational definition

Extended postpartum period: a 12-month period after a live birth.

Postpartum women: women who had live births within the past 1 year prior to date of data collection.

Postpartum family planning use: When a postpartum woman reported using any family planning methods (pills, intrauterine device, injectable, condom, sterilization, or implants), or traditional (breastfeeding or calendar methods) during the 12-month following her most recent childbirth.

### Ethical considerations

Ethical clearance was obtained from the Institutional Review Board (IRB) of the Institute of Public Health, the University of Gondar. Permission letters were obtained from Addis Ababa city and Kolfe Keranyo subcity administrations respectively. Participants were informed about the objectives of the study and reassured about the confidentiality of the findings. A written consent was obtained from each participant.

### Data processing and analysis

The data were checked for completeness and coded manually. EPI-INFO version 7 and SPSS version 20 were used for data entry and analysis, respectively. Descriptive statistics, such as frequencies and percentages were computed to describe the study population in relation to relevant variables. Bivariate and Multivariable logistic regression analyses were carried out to see the presence of association between dependent and the independent variables. Variables with *p*-values of < 0.2 in the Bivariate analysis were further fitted to multivariable logistic regression analysis. Adjusted odds ratios with 95% confidence intervals were computed and variables with *p-* values of < 0.05 in the multivariable analysis were considered as statistically significant.

## Results

### Socio-demographic characteristics

In this study, 803 postpartum women participated with a response rate of 94.9%. Majority of respondents, 675(84.1%), were aged 20–34 years. Regarding respondents marital status, religion, and occupation, 748(93.2%) were married, 454(56.5%) were Orthodox Christians, and 468(58.3%) were housewives. Concerning their educational status, 90(11.1%) of the respondents did not have any formal education, and one-fourth of them, 197(24.5%) were grade 12 and above (Table 1).

**Table 1:** Socio-demographic characteristics of women in the first year of postpartum period in Kolfe Keranyo sub city, Addis Ababa, 2015

Variables		Frequency	Percent
Age			
	15–19	16	2.0
	20–34	675	84.1
	35–49	112	13.9
Educational status			
	No formal education	89	11.1
	Primary	275	34.2
	Secondary	242	30.1
	Above Secondary	197	24.5
Marital status			
	Married	748	93.2
	Unmarried	55	6.8
Ethnicity			
	Amhara	321	40.0
	Oromo	178	22.2
	Tigre	70	8.7
	Gurage	142	17.7
	Siltie	51	6.4
	Other	41	5.0
Occupation			
	Housewife	468	58.3
	Merchant	84	10.5
	Daily Labourer	22	2.7
	Gov/ Private Employee	195	24.3
	Other	34	4.2
Religion			
	Orthodox	454	56.5
	Catholic	31	3.9
	Protestant	109	13.6
	Muslim	209	26.0
Spouse’s educational status (773)			
	No formal education	18	2.2
	Primary	258	258
	Secondary	210	210
	Above Secondary	287	287
Spouse’s occupation(773)			
	Merchant	256	33.1
	Daily Labourer	160	20.7
	Gov/ Private Employee	245	31.7
	Other	112	14.5

### Fertility and reproductive characteristics

Among the respondents, 535(66.6%) had one or two children and about half, 401(49.9%), had a lapse of 6 months since their delivery. The majority, 719(89.5%), reported that their recent pregnancy was planned, and 652(81.2%) desired to have a birth interval of more than 2 years. More than half, 442(55.0%), of the respondents said that menses had not resumed after their recent birth, and 748(93.2%) reported they were breastfeeding at the time of the survey. Slightly more than three-fourths, 629(78.3%), had histories of family planning use before their last pregnancies (Table 2).

**Table 2:** Fertility and reproductive characteristics of women in the first year of postpartum period in Kolfe Keranyo sub city, Addis Ababa, 2015

Variables	Frequency		Percent
Parity			
	1–2	535	66.6
	3–4	243	30.3
	≥ 5	25	3.1
Duration of month since delivery			
	< 6 Months	271	33.7
	6 Months	131	16.3
	> 6 Months	401	49.9
Planned birth			
	Yes	719	89.5
	No	84	10.5
Preferred birth space			
	≤ 2 Years	17	2.1
	> 2 Years	652	81.2
	I Don’t Know	134	16.7
Number of children wish to have			
	≤ 4	443	55.2
	> 4	46	5.7
	I Don’t Know	314	39.1
Health education about FP during ANC			
	Yes	365	45.5
	No	438	54.5
Menses before pregnancy			
	Regular	676	84.2
	Irregular	127	15.8
Menses after delivery			
	Yes	361	45.0
	No	442	55.0
Visit HC after delivery			
	Yes	649	80.8
	No	154	19.2
Currently breastfeeding			
	Yes	748	93.2
	No	55	6.8
Number of FP you know			
	≤ 4	328	40.8
	≥ 4	475	59.2
History of FP use before current pregnancy			
	Yes	629	78.3
	No	174	21.7

### Postpartum family planning method use

**Fig. 1:**
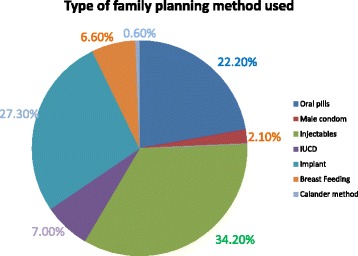
Type of family planning methods used by women in the first year postpartum period in Kolfe Keranyo sub city, Addis Ababa, Ethiopia, 2015

In this study, the prevalence of PPFP use was 80.3% (95% CI: 74.5, 83.1) and the most preferable method used by 221(32.2%) of the women was the injectable (Fig. 1).

### Factors associated with postpartum family planning use

The result of the multivariable analysis showed that marital status, length of time after delivery, menses resumption after recent birth, and history of family planning use before current pregnancy were significantly associated with PPFP use. Unmarried women were 91.0% less likely to use family planning methods as compared to married ones (AOR = 0.09, 95% CI: 0.03, 0.22). Women with a time lapse of over 6 months since their delivery were two times more likely to use family planning method as compared to women who had less than that (AOR = 2.37, 95% CI: 1.18, 4.75). Women who had had menses resumption after recent birth were two times more likely to use family planning method than who had not (AOR = 2.12, 95% CI: 1.37, 3.41). Women who had no history of contraceptive use before their last birth were 88% less likely to use family planning method during the postpartum period compared to those who had (AOR = 0.12, 95% CI: 0.07, 0.18) (Table 3).

**Table 3:** Multivariate analysis showing factors associated with PPFP use in Kolfe Keranyo sub city, Addis Ababa, 2015

Variables		PPFP use		COR 95% CI	AOR 95%CI
		Yes Number (%)	No Number (%)		
Age in years					
15–19		10(62.5)	6(37.5)	1	1
20–34		402(59.6)	273(40.4)	1.13(0.84,2.01)	0.30(0.20,4.70)
35–39		72(64.3)	40(35.7)	0.93(0.45,1.70)	0.61(0.70,3.73)
Marital status					
Married		627(83.3)	121(16.2)	1	1
Unmarried		18(32.7)	37(67.3)	10.65(0.96,11.01)	0.09(0.03,0.22)*
Educational Status					
No formal education		26(29.2)	63(70.8)	1	1
Primary		116(42.2)	159(57.8)	0.57(0.42,2.00)	1.72(0.53,5.49)
Secondary		194(80.2)	48(19.8)	0.10(0.01,3.79)	0.68(0.98,2.40)
Above Secondary		145(73.6)	52(26.4)	0.15(0.11,4.50)	1.60(0.85,3.02)
Time in months since delivery					
<6		192(70.8)	79(29.2)	1	1
6		107(81.7)	24(18.3)	0.55(0.24,1.39)	2.38(1.19,4.80)*
>6		346(86.3)	55(13.7)	0.39(0.17,0.82)	2.73(1.73,4.52)*
Occupation					
House wife		157(33.6)	311(66.4)	1	1
Merchant		43(51.2)	41(48.8)	0.48(0.22,1.59)	1.40(0.91,3.52)
Daily laborer		8(36.4)	14(63.6)	0.88(0.21,3.01)	1.30(0.72,3.03)
Government/Private employee		135(69.2)	60(30.8)	0.22(0.10,1.51)	0.83(0.61,4.50)
Other^a^		8(23.5)	26(76.5)	1.64(0.38,1.20)	1.51(0.87,2.12)
History of previous FP use					
Yes		557(88.6)	72(11.4)	1	1
No		88(50.6)	86(49.4)	7.56(0.95,8.01)	0.12(0.07,0.18)*
Menses resumption after delivery					
Yes		266(73.7)	95(26.3)	1	1
No		379(85.7)	63(14.3)		2.07(1.38,3.41)*

*P < 0.05 = Significant ^a^House servants, Jobless

## Discussion

Postpartum period is an entry point to initiate family planning methods for mothers, but usually it is a missed opportunity. The prevalence of postpartum family planning method (PPFP) use in this study was 80.3% (95% CI: 74.5, 83.1%). This finding is in line with that of a study conducted in Nichisti District Hospital, Malawi where the prevalence of PPFP was 75% [25]. This similarity might be due to the similarity of participants in the two studies in some socio demographic characteristics. For instance, the proportion of women who were married in this study was 93.2%, and in the Malawi study it was 93.3% [25]. Moreover, the educational status of the two participants was almost similar.

Postpartum contraceptive use in this study was higher than those of other studies conducted in Ethiopia, for example the 2011 EDHS, Gondar town, Dabat, Axum, and Somali region reported 55, 48.4, 10.3, 48, and 12.3%, respectively [11, 14, 26–29]. It was also higher than the findings of studies conducted in Uganda (28%) [28] and Rural Uganda (25.0%) [30].

The discrepancy could be due to time gap of studies and the presence of some dissimilar socio-demographic and reproductive characteristics among participants. For instance, literature documented that educational level has a direct relationship with PPFP use [11, 13, 31, 32]. The possible reason for the difference in the prevalence of PPFP might be the difference in educational level of study participants. For example, in the study conducted in Gondar town, the proportion of women who did not have any formal education was higher (21.9%) than that of this study (11.1%). The same was true with the study conducted at Dabat district in which the proportion of women who did not have formal education was 64.4% [26], higher than what was seen in this study. Meanwhile, the proportion of participants with tertiary education in this study was higher (24.5%) than that of the study conducted in Gondar (20.6%) [14]. The proportion of study participants who had higher education was lower in the studies conducted in Dabat, Ethiopia (1.8%) [26] and Axum (12.2%) [27] than that of this study (24.5%). The proportion of women who had secondary education and above was higher (54.6%) in this study compared to (23.7%) of Uganda [28] and 42.6% of rural Uganda [30].

The other possible reasons for the differences between this work and the study done in Axum might be variations in spousal educational status and birth intention, not only women’s educational level, but also that of their husbands can take part in PPFP use. If spouses are educated, they can understand the benefits of having adequate space between births and encourage and advise on the use of family planning methods, which could contribute to the uptake of PPFP.

The proportion of partners educational status in this study was higher than that of Axum. The proportion of mothers who wanted to have birth intervals of less than or equal to 2 years was higher among the participants in Axum (24.2%) than in this study (2.1%) [27].

Marital status of women might have contributions to the observed differences in the prevalence of PPFP use. If a woman is married, she may have early postpartum sexual contact than those who are not married. So, there may be differences in risk perception between the two groups of women that risk perception relating to unwanted or mistimed pregnancy is expected to be high among married women than none married ones. The proportion of our participants who were married was higher (93.2%) than that of the study done in Uganda [28]. Another possible reason for the difference in PPFP use among postpartum women might be differences in perinatal service utilization. Women who had history of antenatal and postnatal care visits might have better chances of getting counselling about contraceptive use. The proportion of mothers who had postnatal care visits was higher in this study (80.8%) than the study done in Dabat, Ethiopia (5.7%) [26].

However, the uptake of PPFP in this study was lower than those of studies conducted in Kenya, Nairobi (95.2%) and South Africa (89.0%) [21, 33]. The difference could be due to the presence of socio-economic differences, cultural variations, and service accessibility.

Unmarried women were 91.0% less likely to use PPFP methods than married ones. This could be due to the fact that unmarried women may be less likely to be sexually active than married ones which might reduce their demand and use of PPFP methods. It might also be explained in terms of the fact that married women might have more access to different PPFP methods compared to unmarried ones.

In this study, women whose menses resumed after the recent birth were two times more likely to use the PPFP method than women whose menses did not (AOR 2.22, 95% CI: 1.39, 3.51). This finding was in line with those of studies conducted in Gondar town and Axum Ethiopia, and in Malawi [14, 16, 27]. That is because most women may tend to believe that the risk of pregnancy is linked to only menses resumption, and might not take family planning methods during the postpartum period.

Duration in months after delivery was found to have a significant association with the use of PPFP. The longer the duration after delivery, the better the use of contraceptives. This finding was similar with the results of studies conducted in Gondar town and Somali region [14, 29]. The possible explanation could be that as the duration of postpartum increased the proportion of women who start sexual activity raise hence, women might suspect pregnancy during the sexual exercise, and decide to use PPFP.

Women who had history of family planning method use prior to their last pregnancy were also found to use contraceptives in their postpartum period more than those who had no such history. This result was similar with that of a study conducted in rural Uganda, where women who had previous history of family planning method use were nearly two times more likely to use family planning methods compared to their counter parts [30]. This could be explained by the fact that women who had history of previous family planning method use might have more knowledge, better attitude, and practice regarding the use of family planning methods compared to those who had not.

The proportion of women who had no history of previous contraceptive use was 88% less likely to use PPFP use than those who had previous experience. This might be explained by the fact that women who had previous history of contraceptive use might have better attitude and practice with regard contraceptives as compared to those who had not.

Since this was a cross-sectional study, it shares the limitations of the study design. Including women within the first 6 weeks postpartum was one of the shortcomings of this study. Moreover, as the study mainly focussed on individual level factors, it is recommended that researchers include factors relating to the health system and service providers in the future.

## Conclusion

This study found that the prevalence of postpartum family planning use was high. Marital status (marriage), number of months after delivery, history of menses resumption after recent birth, and history of family planning use before the current pregnancy were factors significantly associated with postpartum contraceptive use.

## Abbreviations

ANC: Antenatal Care
AOR: Adjusted Odds Ratio
CI: Confidence Interval
COR: Crude Odds Ratio
DHS: Demographic and Health Survey
FP: Family Planning
HC: Health Centre
OR: Odds Ratio
PPFP: Postpartum Family Planning
RTT: Research and Technology Transfer Core Process
SPSS: Statistical Package for the Social Sciences
WHO: World Health Organization
